# Assessment of environmental contamination with *Echinococcus* spp. through DNA detection in free-roaming canid feces and soil in human echinococcosis hotspots from the Three-River-Source Region of the Qinghai-Tibet Plateau, China

**DOI:** 10.1186/s13071-026-07369-2

**Published:** 2026-03-23

**Authors:** Xueyong Zhang, Zhi Li, Yong Fu, Yijuan Ma, Xiuying Shen, Hong Duo, Zhihong Guo, Yadong Zheng, Yingna Jian

**Affiliations:** 1 The Academy of Animal and Veterinary Sciences, Qinghai University, Qinghai Provincial Key Laboratory of Pathogen Diagnosis for Animal Disease and Green Technical Research for Prevention and Control, Xining, 810016 Qinghai China; 2 Key Laboratory of Applied Technology on Green-Eco-Healthy Animal Husbandry of Zhejiang Province, Zhejiang Provincial Engineering Laboratory for Animal Health Inspection and Internet Technology, Zhejiang International Science and Technology Cooperation Base for Veterinary Medicine and Health Management, Belt and Road International Joint Laboratory for One Health and Food Safety, College of Animal Science and Technology and College of Veterinary Medicine of Zhejiang A&F University, Hangzhou, 311300 Zhejiang China

**Keywords:** *Echinococcus* spp., Environmental contamination, Soil and feces samples, Real-time PCR, Three-River-Source Region of the Qinghai-Tibet Plateau

## Abstract

**Background:**

The Three-River-Source Region of the Qinghai-Tibet Plateau is a hyperendemic focus for echinococcosis, with *Echinococcus granulosus*, *E. multilocularis*, and *E. shiquicus* circulating between definitive canid hosts (dogs and foxes) and intermediate hosts (livestock and rodents). However, the extent of environmental contamination by *Echinococcus* eggs remains understudied and poses significant risks to human and animal health.

**Methods:**

From 2019 to 2021, we collected 631 canid fecal samples (296 from dogs and 335 from foxes) and 398 adjacent soil samples across endemic counties in the Three-River-Source Region of the Qinghai-Tibet Plateau. Multiplex real-time PCR was employed to detect *Echinococcus* species DNA in feces and soil samples.

**Results:**

The overall *Echinococcus* prevalence in canid feces was 7.13% (45/631), with 3.01% in *E. multilocularis* (19/631), 2.06% in *E. granulosus* (13/631), and 2.06% in *E. shiquicus* (13/631). Foxes presented increased *E. multilocularis* (3.88%, 13/335) and *E. shiquicus* (2.69%, 9/335) infections, whereas dogs presented increased *E. granulosus* prevalence (2.70%, 8/296). Soil contamination with *Echinococcus* species was detected in 2.51% (10/398) of the samples. The primary contaminants were *E. multilocularis* and *E. shiquicus* (1.01% each, 4/398), whereas *E. granulosus* was less frequent (0.50%, 2/398). Moreover, the soil near fox feces was contaminated with both *E. multilocularis* and *E. shiquicus*, whereas the dog-associated soil was contaminated with all three species.

**Conclusions:**

This study suggests widespread environmental deposition of *Echinococcus* eggs on the Qinghai-Tibet Plateau, driven by canid defecation. If these eggs remain viable, their persistence in soil would indicate a potential zoonotic transmission risk, highlighting the need for integrated control strategies targeting both domestic and wild canids.

**Graphical abstract:**

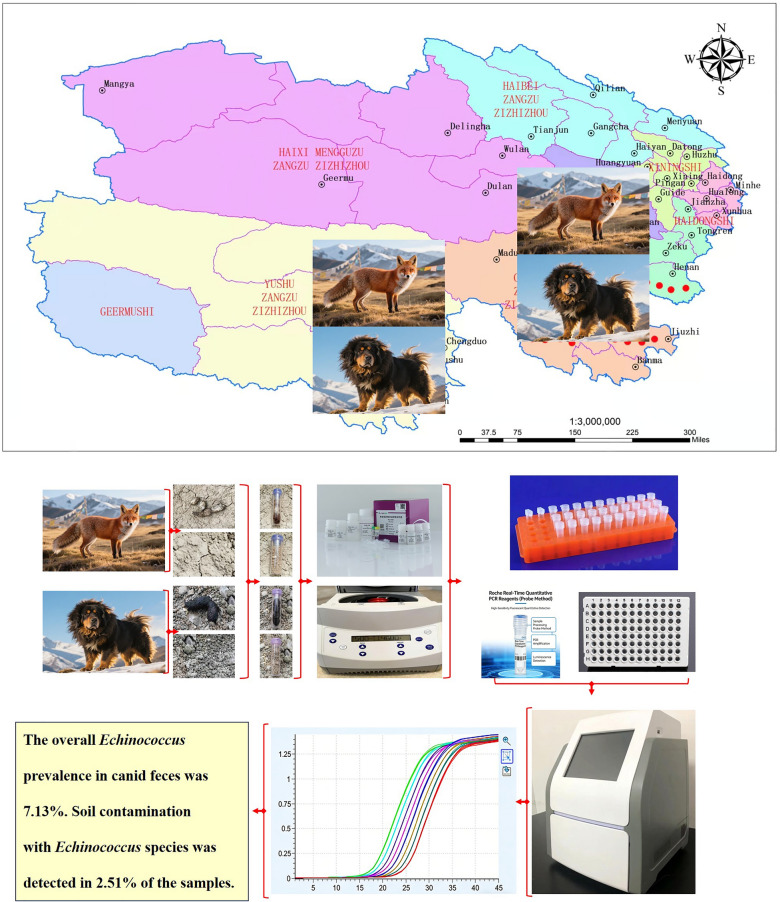

**Supplementary Information:**

The online version contains supplementary material available at 10.1186/s13071-026-07369-2.

## Background

Echinococcosis, also known as hydatid disease, is a significant zoonotic disease caused by the larval stages of *Echinococcus* species, particularly *Echinococcus granulosus* and *Echinococcus multilocularis*, and poses substantial health risks to public health [1]. In humans and animals, infection with *E. granulosus* and *E. multilocularis* leads to cystic echinococcosis (CE) and alveolar echinococcosis (AE), respectively [1]. CE is a global epidemic, whereas AE is predominantly endemic to Northwest China and Central European latitudes of the Northern Hemisphere [1, 2]. Accordingly, echinococcosis has been prioritized by the World Health Organization (WHO) as a neglected tropical disease and as one of the most significant foodborne zoonoses based on its substantial burden worldwide [3]. In Qinghai Province, a province-wide screening across 43 counties (cities and districts) reported a B-ultrasound detection rate of 1.10% in the general population and 0.53% in primary school students, while the seroprevalence (ELISA) was 5.92% [4]. Moreover, regional disparities are evident, with a B-ultrasound prevalence of 0.78% and a seroprevalence of 4.26% in students in Yushu Prefecture [5]. In contrast, Guoluo Prefecture reported a higher B-ultrasound detection rate of 2.9% and a markedly higher seroprevalence of 16.1% in the student population [6].

The Three-River-Source Region on the Qinghai-Tibet Plateau represents a hyperendemic focus for three *Echinococcus* species: *E. granulosus*, *E. multilocularis*, and *E. shiquicus* [7, 8]. The definitive hosts of *Echinococcus* are typically canids such as foxes, dogs, and wolves, whereas intermediate hosts include livestock (cattle and sheep) and small mammals such as voles and pikas, with humans serving as accidental hosts [2, 9]. Between 2007 and 2011, serological surveys in Qinghai via rapid tests revealed *Echinococcus* infection rates of 22.57% in dogs and 13.9% in foxes [10]. More recent local investigations revealed a high prevalence: the fecal antigen positivity rate for *Echinococcus* spp. in dogs from Huangnan Prefecture (covering four counties) was 11.70% [11]; the overall positive rate was 6.35% (16/252) in canid feces in Maqin County, with 3.73% (6/161) in foxes, 6.67% (2/30) in wolves, and 13.11% (8/61) in dogs [12].

In the life cycle of *Echinococcus* spp., following the excretion of feces by definitive hosts, eggs are released into the environment. A substantial portion of this contaminated fecal matter is deposited directly onto grassland soils [13, 14]. Eggs are highly resilient and can persist in the environment for extended periods; they are disseminated with feces, leading to widespread soil contamination and posing a continuous risk to intermediate hosts [15, 16]. Consequently, soil acts as a critical vector for zoonotic parasites, including helminths (via infective eggs) and protozoa (via infective oocysts) [17, 18]. The specific soil properties, high humidity, and high-altitude conditions of the Three-River-Source Region are particularly conducive to the dispersal and prolonged survival of these parasitic pathogens [8, 19]. For example, under cold and moist conditions, *Echinococcus* eggs can remain viable for > 1 year [15, 20, 21]. In addition, behaviors such as hand-to-mouth contact with soil contaminated with feces or direct geophagia (soil ingestion) have been consistently identified as significant risk factors for the development of soil-transmitted parasitic diseases [22, 23]. Consequently, contaminated soil, particularly in and around the dens of definitive canid hosts, represents a significant environmental reservoir and a growing focus of public health concern [24]. Despite this recognized risk, public awareness of the threats posed by zoonotic parasites originating from the intestinal tracts of wild and domestic carnivores remains critically low [25]. Furthermore, scientific research specifically quantifying parasitic contamination levels in the soil surrounding carnivore dens remains scarce, leaving a key knowledge gap in understanding the complete transmission cycle and in developing effective exposure mitigation strategies [15, 26].

The red fox (*Vulpes vulpes*) is the primary definitive host for *E. multilocularis* within the Three-River-Source Region, resulting in severe environmental *Echinococcus* egg contamination in forested wildlife habitats [8]. Juvenile foxes, which harbor high parasite biomass and exhibit dispersal behaviors, represent a high-risk demographic for the spatial dissemination of *Echinococcus* pathogens [26, 27]. Although the prevalence of *Echinococcus* infection in domestic dogs (particularly Tibetan mastiffs in Tibetan pastoral areas) is relatively low, their high population density, frequent close contact with humans, and high reproductive potential of parasites within canine hosts establish them as crucial transmitters for both *E. granulosus* and *E. multilocularis*, thereby playing a significant role in the local transmission cycle of echinococcosis [15, 28, 29]. Humans, as dead-end intermediate hosts, cannot participate in the transmission cycle after infection, but the severe health risks we face cannot be ignored [30]. Domestic dogs play a central role in the transmission of echinococcosis through direct contact with humans or by contaminating soil, water sources, and food crops [24, 29]. Studies have shown that tapeworm eggs can persist for extended periods in the natural environment [16], with *Echinococcus* spp. eggs maintaining viability and infectivity for up to several months to years under favorable conditions [2, 31]. The substantial burden of proglottids and eggs excreted in canine feces, combined with their pronounced environmental resilience, underscores the severe threat of environmental pollution to public health [15, 29, 32].

Increasing amounts of epidemiological data from the Three-River-Source Region indicate persistently high infection rates of *Echinococcus* spp. among both intermediate and definitive hosts [8, 33]. This confirms that active transmission cycles are maintained in the region, posing a continuous risk and presenting a serious threat to the health of local farmers and herders, as well as to the economic sustainability of animal husbandry [28]. While numerous studies have assessed infection prevalence in definitive [8, 26, 34] and intermediate hosts, including humans [35, 36] and livestock [37–39], a significant knowledge gap remains regarding the transmission dynamics of eggs in the environment. Although the public health challenges of echinococcosis are widely acknowledged [1, 30], the risks of human exposure to *Echinococcus* spp. originating from environmentally disseminated eggs in the Three-River-Source Region have not been systematically evaluated. To address this, in the present study, fecal samples were collected from canid hosts (foxes and dogs) at representative locations across the region, alongside soil samples from areas frequented by these animals. This study aims to provide a preliminary assessment of the extent of *Echinococcus* egg contamination in both canid feces and soil, thereby providing essential baseline data for future environmental risk analyses of echinococcosis in this ecologically fragile and endemic zone.

## Methods

### Study areas and samples

This study was conducted in the Three-River-Source Region of the Qinghai-Tibet Plateau, China, between April 2019 and October 2021. Located in the southern part of Qinghai Province, this region (31°39'–36°16'N, 89°24'–102°23'E) covers an area of approximately 363,000 km² within the hinterland of the Qinghai-Tibet Plateau. It encompasses 16 counties distributed across four Tibetan autonomous prefectures: Yushu, Guoluo, Hainan, and Huangnan. The region has a total population of approximately 550,000 inhabitants, predominantly of Tibetan ethnicity, with a population density of approximately 1 person per km² (according to the official website of the Qinghai Provincial Bureau of Statistics). Livestock inventories consist of approximately 4 million yaks and 3.5 million Tibetan sheep. With an average elevation ranging from 4000 to 4800 m, the Three-River-Source region represents one of the world’s highest and most extensive plateau wetland ecosystems and is characterized by alpine mountain ranges, glaciers, permafrost, broad valleys, basins, and numerous lakes and wetlands (according to the official website of the Department of Natural Resources of Qinghai Province and Three-River-Source National Park Administration).

**Fig.1:**
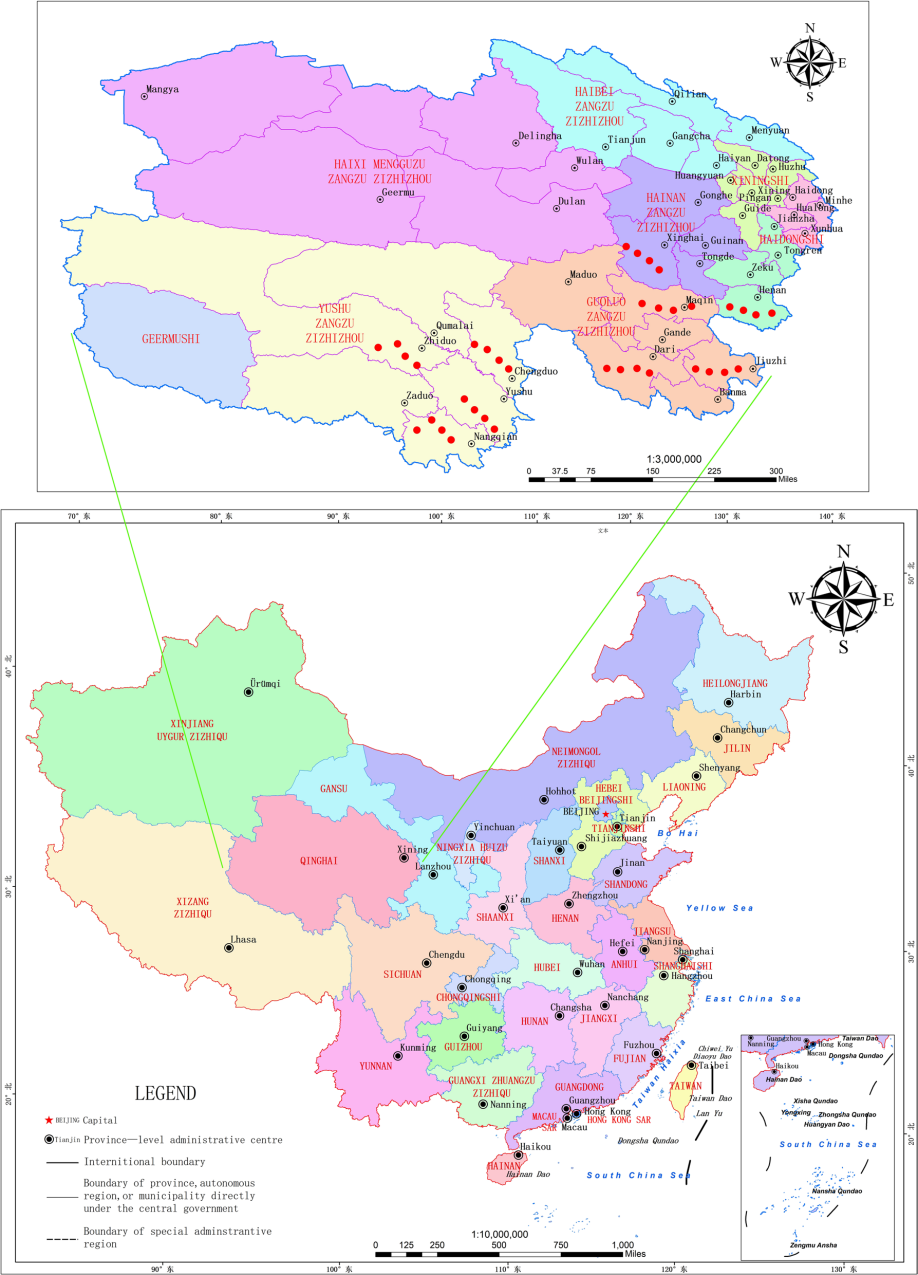
Map of the study sites

The sampling sites were identified through a systematic review of epidemiological reports on echinococcosis in humans and canids in the Three-River-Source region. High-risk endemic areas were prioritized, focusing on counties with documented infections in both humans and domestic dogs, including Chenduo, Dari, Xinghai, Maqin, Jiuzhi, Henan, Yushu, Nangqian, and Zhiduo. Emphasis was placed on hyperendemic zones exhibiting high-risk ecological characteristics, including (i) high densities of domestic and stray dogs coupled with frequent wild fox activity (definitive hosts); (ii) intensive livestock grazing (intermediate hosts) and abundant small rodent populations; (iii) geographical attributes such as dense river networks and irrigated pastures; (iv) vegetation-rich environments near rivers, which have been associated with disease hotspots (Fig. 1 and Additional file 1: Table S1 and Additional file 2: Table S1).

Sampling was conducted with the direct guidance of local veterinary authorities. We specifically targeted and collected samples from discrete locations within these counties identified as high risk based on the four ecological features. The sampling sites were therefore not intended to be geographically representative of the entire county but were selected precisely because they embodied the high-risk ecologies under investigation. A total of 631 canid fecal samples were collected. Fresh fecal samples from canids were aseptically collected using sterile tongs and disposable sampling bags, in accordance with a strict one-sample-per-individual protocol. Fox feces (*n* = 335) were exclusively collected in the vicinity of active den sites, which were typically located far from nomadic settlement tents and were confirmed to have recent signs of fox activity (e.g., fresh footprints, frequent sightings). In contrast, dog feces (*n* = 296) were primarily gathered in and around residential areas. This targeted approach ensured accurate host source attribution based on ecological and behavioral context. Adjacent soil samples were collected from within a 0.5–1-m radius of fecal deposits, with a standardized quantity of approximately 100 g per sample. Sampling specifically targeted areas of visible fecal contamination, such as soil surrounding fragmented feces or that was influenced by long-retained, dried feces (though the dried fecal matter itself was not collected). Following identical protocols in both dog and fox habitats, 398 samples were obtained, excluding locations that were logistically inaccessible. All samples were meticulously labeled, cataloged, and cryopreserved at − 80 °C until subsequent molecular analysis.

### Sample processing and genomic DNA extraction

Helminth eggs (assumed existing) were enriched from soil and fecal samples (50 g each) via a sedimentation-based protocol. The samples were homogenized in a glass container with distilled water to form a uniform suspension, which was then filtered through a 40–60-mesh stainless steel sieve into a 500-ml graduated conical cylinder. After gravitational sedimentation for 30 min, the supernatant was carefully decanted. The resulting sediment was subjected to 3–5 washing cycles with equal volumes of distilled water until the supernatant appeared clear. This step removed free DNA and degraded products from the fecal and soil matrices, thereby ensuring that subsequent PCR signals were derived from intact helminth eggs' genomic DNA. The final pellet was collected for downstream DNA extraction.

Genomic DNA was isolated from the enriched material via the TIANGEN Stool Genomic DNA Extraction Kit (DP328, TIANGEN Biotech, China) in strict accordance with the manufacturer’s instructions. The purified DNA was quantified via spectrophotometry, aliquoted, and stored at – 20 °C until further molecular analysis.

### Molecular analysis

Genomic DNA extracted from fecal and soil samples was analyzed via a multiplex real-time quantitative PCR (qPCR) assay targeting the mitochondrial *cox1* gene fragments of *E. multilocularis*, *E. granulosus*, and *E. shiquicus* [40]. Species-specific probes and universal primer pairs were employed (Table 1). Sample positivity was determined based on explicitly defined criteria, including cycle threshold (Ct) cut-offs and consistent detection in duplicate runs, as specified in the Methods. To control false-negative results, an internal inhibition control was included in each reaction. Technical replicates were performed for all samples, and final classification required concordant results across replicates. Furthermore, a subset of qPCR-positive samples underwent microscopic examination to assess the presence of morphologically consistent taeniid eggs. The consolidated qPCR datasets were subjected to rigorous statistical analysis via predefined frameworks to evaluate the spatial and temporal patterns of the *Echinococcus* species DNA positivity rate across different sample types.

**Table 1:** Sequences of primers and probes used for multiplex real-time PCR for *Echinococcus* spp.

*Cox*1gene primers and probes	Sequences (5′-3′)	GenBank no.
Forward primer	F: TTGTTTGCTATGTTTTCTATAGTGT	NC000928/NC044548/NC009460
Reverse primer	R: TCTTCACATCYAACCCAACAGT	NC000928/NC044548/NC009460
*Echinococcus multilocularis* probe	FAM-TTTAGGGAGTAGTGTTT-MGB	NC000928
*Echinococcus granulosus* probe	VIC-TTTGGGTAGCAGGGTT-MGB	NC044548
*Echinococcus shiquicus* probe	CY5-TTTAGGAAGGAGAGTTT-MGB	NC009460

### Statistical analysis

The detection DNA positivity rates of *Echinococcus* spp. in canine and fox fecal and soil samples were expressed as positivity percentages with 95% confidence intervals (CIs). All statistical analyses followed established epidemiological reporting standards to ensure robust interpretation of spatial distributions and host-associated patterns of parasite transmission.

## Results

During a 30-month surveillance period (2019–2021), 631 fresh canid fecal samples were systematically collected across 10 georeferenced sites within the Three-River-Source Region of the Qinghai-Tibet Plateau. The samples were stratified into 296 from free-roaming dogs and 335 from foxes. This design was further complemented by the collection of 398 spatially paired soil samples, each within a 1-m radius of the fecal deposition site. All the sample metadata are summarized in Tables 2, 3, 4, and 5.

**Table 2:** The detection results of *Echinococcus*. spp DNA in feces samples from free-ranging feral domestic dogs in Three-River-Source Region

Sampling sites	Number of samples	Number of positive samples (%/95% CI)			Total number of positive samples (%/95% CI)
		*Echinococcus multilocularis*	*Echinococcus granulosus*	*Echinococcus shiquicus*	
Chenduo	28	1 (3.57, 0.63–17.71)	0 (0, 0.00–12.06)	0 (0, 0.00–12.06)	1 (3.57, 0.63–17.71)
Dari	101	4 (3.96, 1.55–9.74)	7 (6.93, 3.40–13.62)	0 (0, 0.00–3.66)	11 (10.89, 6.19–18.46)
Xinghai	25	0 (0, 0.00–13.32)	0 (0, 0.00–13.32)	0 (0, 0.00–13.32)	0 (0, 0.00–13.32)
Maqin	15	0 (0, 0.00–20.39)	0 (0, 0.00–20.39)	0 (0, 0.00–20.39)	0 (0, 0.00–20.39)
Jiuzhi	25	0 (0, 0.00–13.32)	0 (0, 0.00–13.32)	0 (0, 0.00–13.32)	0 (0, 0.00–13.32)
Henan	13	0 (0, 0.00–22.81)	0 (0, 0.00–22.81)	0 (0, 0.00–22.81)	0 (0, 0.00–22.81)
Yushu	31	0 (0, 0.00–11.02)	0 (0, 0.00–11.02)	0 (0, 0.00–11.02)	0 (0, 0.00–11.02)
Nangqian	31	0 (0, 0.00–11.02)	0 (0, 0.00–11.02)	4 (12.90, 5.14–28.85)	4 (12.90, 5.14–28.85)
Zhiduo	27	1 (3.70, 0.66–18.28)	1 (3.70, 0.66–18.28)	0 (0, 0.00–12.45)	2 (7.41, 2.06–23.37)
Total number of positive samples (%/95% CI)	296	6 (2.03, 0.93–4.35)	8 (2.70, 1.38–5.24)	4 (1.35, 0.53–3.42)	18 (5.41, 3.35–8.60)

**Table 3:** Detection results of *Echinococcus*. spp DNA in feces samples from free-ranging foxes in the Three-River-Source Region

Sampling sites	Number of samples	Number of positive samples (%/95% CI)			Total number of positive samples (%/95% CI)
		*Echinococcus multilocularis*	*Echinococcus granulosus*	*Echinococcus shiquicus*	
Chenduo	42	4 (9.52, 3.77–22.06)	0 (0, 0.00–8.38)	0 (0, 0.00–8.38)	4 (9.52, 3.77–22.06)
Dari	151	9 (5.96, 3.17–10.94)	5 (3.31, 1.42–7.52)	2 (1.32, 0.36–4.70)	16 (10.60, 6.63–16.52)
Xinghai	49	0 (0, 0.00–7.27)	0 (0, 0.00–7.27)	0 (0, 0.00–7.27)	0 (0, 0.00–7.27)
Maqin	18	0 (0, 0.00–17.59)	0 (0, 0.00–17.59)	0 (0, 0.00–17.59)	0 (0, 0.00–17.59)
Jiuzhi	6	0 (0, 0.00–39.03)	0 (0, 0.00–39.03)	0 (0, 0.00–39.03)	0 (0, 0.00–39.03)
Henan	0	0	0	0	0
Yushu	64	0 (0, 0.00–5.66)	0 (0, 0.00–5.66)	7 (10.94, 5.40–20.90)	7 (10.94, 5.40–20.90)
Nangqian	2	0 (0, 0.00–65.76)	0 (0, 0.00–65.76)	0 (0, 0.00–65.76)	0 (0, 0.00–65.76)
Zhiduo	3	0 (0, 0.00–56.15)	0 (0, 0.00–56.15)	0 (0, 0.00–56.15)	0 (0, 0.00–56.15)
Total number of positive samples (%/95% CI)	335	13 (3.88%, 2.28–6.53)	5 (1.49, 0.64–3.45)	9 (2.69, 1.42–5.03)	27 (8.06, 5.60–11.47)

**Table 4:** Detection results of *Echinococcus*. spp DNA in soil samples adjacent to dogs feces samples in the Three-River-Source Region

Sampling sites	Number of samples	Number of positive samples (%/95% CI)			Total number of positive samples (%/95% CI)
		*Echinococcus multilocularis*	*Echinococcus granulosus*	*Echinococcus shiquicus*	
Chenduo	11	0 (0, 0.00–25.88)	0 (0, 0.00–25.88)	0 (0, 0.00–25.88)	0 (0, 0.00–25.88)
Dari	86	1 (1.16, 0.21–6.30)	2 (2.33, 0.64–8.09)	0 (0, 0.00–4.28)	3 (3.49, 1.19–9.76)
Xinghai	8	0 (0, 0.00–32.44)	0 (0, 0.00–32.44)	0 (0, 0.00–32.44)	0 (0, 0.00–32.44)
Maqin	14	0 (0, 0.00–21.53)	0 (0, 0.00–21.53)	0 (0, 0.00–21.53)	0 (0, 0.00–21.53)
Jiuzhi	15	0 (0, 0.00–20.39)	0 (0, 0.00–20.39)	0 (0, 0.00–20.39)	0 (0, 0.00–20.39)
Henan	15	0 (0, 0.00–20.39)	0 (0, 0.00–20.39)	0 (0, 0.00–20.39)	0 (0, 0.00–20.39)
Yushu	10	0 (0, 0.00–27.75)	0 (0, 0.00–27.75)	0 (0, 0.00–27.75)	0 (0, 0.00–27.75)
Nangqian	28	0 (0, 0.00–12.06)	0 (0, 0.00–12.06)	2 (7.14, 1.98–22.64)	2 (7.14, 1.98–22.64)
Zhiduo	26	1 (3.85, 0.68–18.89)	0 (0, 0.00–12.87)	0 (0, 0.00–12.87)	1 (3.85, 0.68–18.89)
Total number of positive samples (%/95% CI)	213	2 (0.94, 0.26–3.36)	2 (0.94, 0.26–3.36)	2 (0.94, 0.26–3.36)	6 (2.82, 1.30–6.01)

**Table 5:** Detection results of *Echinococcus*. spp DNA in soil samples adjacent to foxes feces samples in the Three-River-Source Region

Sampling sites	Number of samples	Number of positive samples (%/95% CI)			Total number of positive samples (%/95% CI)
		*Echinococcus multilocularis*	*Echinococcus granulosus*	*Echinococcus shiquicus*	
Chenduo	12	0 (0, 0.00–24.25)	0 (0, 0.00–24.25)	0 (0, 0.00–24.25)	0 (0, 0.00–24.25)
Dari	112	2 (1.79, 0.49–6.28)	0 (0, 0.00–3.32)	1 (0.89, 0.16–4.88)	3 (2.68, 0.92–7.58)
Xinghai	13	0 (0, 0.00–22.81)	0 (0, 0.00–22.81)	0 (0, 0.00–22.81)	0 (0, 0.00–22.81)
Maqin	16	0 (0, 0.00–19.36)	0 (0, 0.00–19.36)	0 (0, 0.00–19.36)	0 (0, 0.00–19.36)
Jiuzhi	9	0 (0, 0.00–29.91)	0 (0, 0.00–29.91)	0 (0, 0.00–29.91)	0 (0, 0.00–29.91)
Henan	0	0	0	0	0
Yushu	18	0 (0, 0.00–17.59)	0 (0, 0.00–17.59)	1 (5.56, 0.99–25.76)	1 (5.56, 0.99–25.76)
Nangqian	2	0 (0, 0.00–65.76)	0 (0, 0.00–65.76)	0 (0, 0.00–65.76)	0 (0, 0.00–65.76)
Zhiduo	3	0 (0, 0.00–56.15)	0 (0, 0.00–56.15)	0 (0, 0.00–56.15)	0 (0, 0.00–56.15)
Total number of positive samples (%/95% CI)	185	2 (1.08, 0.30–3.86)	0 (0, 0.00–2.03)	2 (1.08, 0.30–3.86)	4 (2.16, 0.84–5.43)

The qPCR results revealed that 45 canid fecal samples (dogs and foxes) were positive for *Echinococcus* spp., yielding an overall positive rate of 7.13% (45/631). The *Echinococcus* spp. DNA positivity rate was 6.08% (18/296) in dogs and 8.06% (27/335) in foxes. However, no mixed infections, defined as the simultaneous presence of two or more *Echinococcus* species in a single sample, were detected. Among the positive samples, species-specific identification revealed that *E. multilocularis* was the most prevalent species (42.2%, 19/45), followed by *E. granulosus* and *E. shiquicus*, with the same positive rate of 28.9% (13/45).

Among the 19 *E. multilocularis*-positive fecal samples (overall DNA positivity rate: 3.01%, 19/631), foxes presented a higher positive rate (3.88%, 13/335) than dogs (2.03%, 6/296). In contrast, the overall DNA positivity rate of *E. granulosus* was 2.06% (13/631), with dogs showing a higher rate (2.70%, 8/296) than foxes (1.49%, 5/335). Similarly, *E. shiquicus* infections (overall DNA positivity rate: 2.06%, 13/631) were more frequently detected in foxes (2.69%, 9/335) than in dogs (1.35%, 4/296). These findings reveal distinct host preferences among the three *Echinococcus* species in the study area.

Soil analysis revealed 10 *Echinococcus* spp. DNA-positive samples, with a positivity rate of 2.51% (10/398). Among the positive samples, *E. multilocularis* and *E. shiquicus* each accounted for 40.0% (4/10), whereas *E. granulosus* represented 20.0% (2/10). Similarly, no mixed-species contamination was detected. The species-specific environmental contamination rates were 1.01% (4/398) for both *E. multilocularis* and *E. shiquicus* and 0.50% (2/398) for *E. granulosus*. These results indicate environmental contamination by multiple *Echinococcus* species in the surveyed areas.

The soil adjacent to canine fecal deposits presented an overall *Echinococcus* contamination rate of 2.82% (6/213). At the population level, *E. multilocularis*, *E. shiquicus*, and *E. granulosus* each had a low contamination rate of 0.94% (2/213), suggesting similar environmental shedding and persistence across all three *Echinococcus* species in canine-associated microenvironments.

The soils adjacent to the fox fecal deposits presented a lower overall *Echinococcus* contamination rate (2.16%, 4/185), with detections restricted exclusively to *E. multilocularis* and *E. shiquicus*, each representing 50% (2/4) of the positive samples. No *E. granulosus* DNA was detected in this sample set. The population-level contamination rate was 1.08% (2/185) for both *E. multilocularis* and *E. shiquicus*, highlighting the absence of *E. granulosus* in fox-associated soils. These findings suggest distinct host-mediated dissemination patterns, implicating foxes as important contributors to environmental contamination with *E. multilocularis* and *E. shiquicus* in the study region.

## Discussion

This study was designed to evaluate environmental contamination by *Echinococcus* species in the Three-River-Source Region of the Qinghai-Tibet Plateau, an ecologically sensitive and biodiverse area. To address this objective, we systematically collected soil and canine fecal samples from representative sites and performed comprehensive molecular analyses targeting *Echinococcus* species genomic DNA. Our results provide critical empirical evidence regarding the extent and distribution of *Echinococcus* egg contamination in this vulnerable ecosystem, highlighting substantial zoonotic risks for both human and animal populations [1, 2, 19]. The detection of *Echinococcus* DNA in environmental samples is a significant public health concern, as eggs shed by infected canids—including domestic dogs, wolves, and foxes—can be widely disseminated and remain infective in the environment for long periods [41]. Of particular concern is the potential for human exposure through direct contact with contaminated soil or indirect routes such as the consumption of contaminated water and agricultural products [42–44]. Therefore, there is an urgent need to implement integrated One Health approaches to effectively combat echinococcosis in this ecologically fragile region, which combine environmental surveillance, deworming, public health education, and other methods to reduce transmission risks.

Humidity was identified as a significant predictor in univariate models and showed a strong association with CE incidence in multivariate analyses. Elevated humidity—particularly during winter—increases soil moisture content, thereby substantially enhancing the viability and environmental persistence of *Echinococcus* eggs. This prolongs the infectious window and increases the risk of transmission to intermediate hosts and even humans [45]. In support of this observation, relatively high environmental concentrations of *E. granulosus* eggs were observed during humid seasonal periods, notably in winter [46]. A complementary experimental study demonstrated that the viability of *E. granulosus* eggs was highly dependent on relative humidity, with survival rates of 50%, 20%, and 5% at 80%, 60%, and 25% relative humidity, respectively [47]. These findings collectively underscore the critical role of humidity in affecting the transmission dynamics of CE. In this study, the sampling area was characterized by a typical plateau climate, featuring persistently low temperatures and high surface humidity. These specific climatic factors significantly prolong the survival time and maintain the viability of parasitic *Echinococcus* eggs in the environment. Our analytical results demonstrate a high degree of spatial concordance between these conditions conducive to egg persistence and the positive rate of contamination hotspots identified through our analysis (Additional file 1: Table S1 and Additional file 2: Table S1). This spatial coupling probably leads to the accumulation of contamination pressure in localized areas and, correspondingly, may increase the exposure and infection risks for host animals within these identified hotspots.

Our results demonstrate the successful simultaneous detection of three *Echinococcus* species in environmental samples—both soil and feces—via real-time PCR. The high sensitivity of this molecular approach enabled accurate detection of *Echinococcus* DNA, providing reliable data on the prevalence and spatial distribution of environmental contamination. As expected, *Echinococcus* DNA was detected across all sample types, with all three species identified in both the soil and fecal samples. To our knowledge, this study represents one of the few dedicated reports focusing on the detection of *Echinococcus* spp. environmental contamination in the Three-River-Source Region of the Qinghai-Tibet Plateau. Nonetheless, the transmission dynamics of *Echinococcus*, particularly temporal fluctuations and interactions with host animal populations, are not fully understood, largely because of the limited temporal and spatial scope of previous epidemiological surveys conducted in the region.

In the present study, the overall *Echinococcus* spp. DNA-positive rate was 6.08% (18/296) in domestic dogs and 8.06% (27/335) in wild foxes. Compared with the prevalence reported in the attached documents, the *Echinococcus* DNA-positive rate in dogs, as well as in wild canids (foxes), is relatively low. This pattern suggests that the control measures implemented for domestic dogs, such as regular deworming and health management, are highly effective [48]. Nevertheless, the persistent detection of *Echinococcus* in foxes, combined with the unavailability of targeted interventions for wild definitive hosts, indicates that the enzootic transmission cycle remains unbroken. Sustained environmental contamination from infected wild canids continues to pose a challenge for the eradication of echinococcosis in the region. It is indeed a limitation of our methodology that qPCR detection of *Echinococcus* DNA cannot distinguish between viable and non-viable eggs. As noted, an important consideration is that the persistence of *Echinococcus* DNA in the environment does not equate to the presence of infectious *Echinococcus* eggs. The qPCR assay confirms the presence of *Echinococcus* DNA but cannot determine egg viability. Consequently, while our data robustly identify contamination hotspots based on DNA positivity, they likely overestimate the true infection risk. The actual risk to susceptible hosts is contingent upon encountering viable eggs and thus may be lower than the distribution of DNA-positive samples implies. Future research employing viability assays would be invaluable to refine these risk assessments.

In recent years, Qinghai Province has established a comprehensive echinococcosis prevention and control program, integrating multiple interventions, including regular deworming of domestic dogs (definitive hosts), vaccination of intermediate host sheep, health education targeting high-risk populations—particularly children, herders, and monastic communities—and safe disposal of dog feces and hydatid cysts [49]. Concurrently, institutional mechanisms have been strengthened to ensure the precise implementation of government-led training, public awareness campaigns, and livestock-oriented prevention strategies [50]. Despite these efforts, current control policies and operational measures do not adequately block *Echinococcus* transmission within wildlife reservoirs, which include both wild definitive hosts (e.g., foxes) and intermediate hosts (e.g., wild rodents) [8, 19, 51]. This gap poses a substantial threat to the long-term efficacy of control efforts, potentially facilitating the persistence of zoonotic cycles in endemic regions.

Surveillance data consistently report high *Echinococcus* infection rates among wild canids—particularly foxes and wolves—in the ecologically sensitive Three-River-Source Region of the Qinghai-Tibet Plateau [7, 10, 33, 51]. Concurrently, populations of these wild definitive hosts are being driven by the establishment of nature reserves, growing public awareness of wildlife conservation, and regional cultural practices. While a positive ecological development, this recovery has precipitated a deeper ecological overlap between wildlife habitats and human-dominated landscapes. Our findings confirm the presence of *Echinococcus* infection in both domestic dogs and wild foxes within the region. More critically, we observed that the transmission pathways—once relatively distinct between the domestic (dog-livestock-human) and sylvatic (wildlife) cycles—are now increasingly interconnected. This convergence facilitates the spillover of *Echinococcus* between cycles, effectively linking transmission among wildlife, livestock, domestic dogs, and humans. Consequently, the frequent integration of wildlife into human activity zones increases the risk of zoonotic transmission and necessitates integrated surveillance and control strategies that encompass both domestic and wild hosts.

The challenges in managing wildlife-based transmission are multifaceted. The extensive home ranges and high mobility of wild canids render conventional control methods, such as fixed-point deworming, largely ineffective. Similarly, interventions targeting abundant and widely distributed wild intermediate hosts, like rodents and pikas, are logistically impractical. Current reliance on sporadic baiting stations is implemented without systematic evaluation, leaving the natural transmission cycle largely unbroken. Furthermore, the absence of a standardized framework for wildlife disease control is exacerbated by administrative fragmentation. Forestry departments, while knowledgeable in wildlife ecology, often lack disease control expertise, whereas veterinary and public health agencies possess the relevant expertise but have limited jurisdiction over wildlife. This misalignment results in uncoordinated and fragmented control efforts.

The consequences of these shortcomings are severe. The persistence of *Echinococcus* in wildlife reservoirs enables continuous environmental contamination with infectious eggs, creating a cyclical “control-rebound” dynamic that undermines elimination efforts. Even with successful domestic dog control, environmental contamination from wildlife can lead to reinfection of livestock and humans. Moreover, the high mobility of wild hosts may facilitate the regional spread of *Echinococcus*, potentially expanding the endemic area and diluting the impact of existing control achievements.

Therefore, achieving sustainable interruption of *Echinococcus* transmission requires a holistic “One Health” strategy. This strategy must integrate advanced surveillance systems—employing satellite tracking, environmental DNA, and remote sensing—to monitor host movements and identify transmission hotspots. Intervention strategies must be intelligent and multi-modal, combining scaled-up domestic dog deworming with the development of long-acting baits and automated bait stations for wild canids. Finally, overcoming administrative barriers is paramount. This requires establishing a robust cross-departmental coordination mechanism involving forestry, agriculture, animal health, and public health sectors and incorporating wildlife disease control into local government performance evaluations to ensure sustained commitment and efficacy.

The findings of this study revealed a soil contamination rate of 2.51% (10/398) with *Echinococcus* DNA. Although this prevalence may appear relatively low, its close association with the activity patterns of definitive hosts underscores its epidemiological significance and warrants serious concern. The unique climatic conditions of the Three-River-Source Region—characterized by extended low-temperature periods—provide an ideal environment for the prolonged survival of *Echinococcus* eggs. Furthermore, the substantial egg output of adult tapeworms, combined with their documented resilience to cold and desiccation, facilitates long-term environmental persistence and widespread dispersal through both fecal matter and soil. These factors collectively contribute to the sustained transmission and prevalence of echinococcosis in this ecologically sensitive region. The detection of *Echinococcus* DNA in soil represents a direct pathway for environmental exposure, increasing infection risk for both human populations and susceptible intermediate host species. This environmental reservoir thereby amplifies the overall zoonotic burden and complicates control efforts. Consequently, soil monitoring should be incorporated as a key component of integrated surveillance and public health strategies.

One of the limitations of this study is the relatively small sample size, which may affect the representativeness of the findings and limit the generalizability of the estimated contamination rates. Additionally, the absence of both “wet-lab” experimental validation and clinical correlation analyses limits our ability to conduct a full, rigorous assessment of the public health impacts associated with environmental *Echinococcus* contamination. Although multiplex fluorescent real-time PCR is sensitive, further sampling and experimental validation are needed to improve the accuracy and reliability of the results. Future studies should prioritize expanding the sample size across diverse ecological and administrative regions and incorporating complementary detection techniques, such as coproantigen ELISA, to better characterize the environmental distribution and infectivity of *Echinococcus* eggs. In addition to ongoing deworming programs targeting domestic dogs, future efforts should focus on understanding the transmission dynamics of *Echinococcus* in wild definitive hosts (e.g., foxes and wolves) and developing feasible intervention strategies for wildlife populations. Such integrated approaches are essential for disrupting the transmission cycle of echinococcosis across human, domestic animal, and wildlife interfaces. Broader environmental sampling and ecological studies will be critical for further elucidating the survival traits of *Echinococcus* eggs in natural environments and the threats to human and animal health.

## Conclusions

This study provides clear evidence that soil ecosystems within the Three-River-Source region of the Qinghai–Tibet Plateau are contaminated with eggs of *Echinococcus* spp. The documented extent of environmental contamination underscores significant public health concerns and highlights the urgent need for targeted strategies to prevent and control the spread of echinococcosis. Based on these findings, several recommendations are proposed. Veterinary and public health authorities should enhance the management of stray dog populations and establish systematic, large-scale deworming programs. Simultaneously, forestry and grassland management departments should prioritize research on the infection dynamics of *Echinococcus* spp. in wild definitive hosts—such as foxes and wolves—and develop feasible intervention measures to reduce environmental contamination from wild animals. At the community level, herdsmen and parents are advised to prevent children from coming into contact or playing with soil in residential areas where dogs are present to minimize exposure risk.

## Supplementary Information

Additional file 1: Table S1. The geographical and climatic conditions of sampling sites in the current study.

Additional file 2: Table S1. The presence and prevalence of *Echinococcus* species in different hosts from different geographic origins from related literature.

## Data Availability

Data supporting the main conclusions of this study are included in the manuscript.
