# Supplementary material for: Assessment of environmental contamination with Echinococcus spp. through DNA detection in free-roaming canid feces and soil in human echinococcosis hotspots from the Three-River-Source Region of the Qinghai-Tibet Plateau, China
Source: Parasit Vectors. 2026 Mar 23;19:192. doi: 10.1186/s13071-026-07369-2 (PMC13130420; doi:10.1186/s13071-026-07369-2)
Supplement: Supplementary file 1 — Additional file 1: Table S1. The geographical and climatic conditions of sampling sites in the current study. [file 13071_2026_7369_MOESM1_ESM.docx]

Additional file 1 Table S1 The geographical and climatic conditions of sampling sites in the current study

| Sample site | Location and overview | Climate conditions (Regional area; Average altitude; Average annual temperature; Average annual precipitation) | Transit rivers and major water sources | Natural and cultural attractions |
| --- | --- | --- | --- | --- |
| Zhiduo | 89°23′~96°23′ E; 33°02′~36°16′ N. Zhiduo County is located in the southwestern part of Qinghai Province, close to Xinjiang Uygur Autonomous Region and Tibet Autonomous Region in the west, bordering to Yushu County in the north, to Qumalai County and Haixi Prefecture in the north, to Zaduo County in the south, there is Kekexili National Nature Reserve within the county, which is one of the most completely preserved areas in the world in terms of pristine ecological environment, is also the largest in area, highest in elevation, and the richest wildlife resources in China. It has a pivotal role in the ecological environment of the Tibetan Plateau, China, even Asia and the world. Zhiduo County is the birthplace of the Yangtze River, and is known as the “First County of the Yangtze River”. | ~ 80600 km^2^  ~4500 m  -0.3 ℃  ~390 mm | Tongtian River (Yangtze River), Dangqu, Yaqu, Cocos Lake, Sun Lake, Xijin Ulan Lake | Cocosili Nature Reserve, Kusai Lake, Dronai Lake, Unfrozen Spring, Gongsa Monastery |
| Nangqian | 95°22'~97°07′ E; 31°32′~32°43′ N. Nangqian County is adjacent to the Haixi Mongolian-Tibetan Autonomous Prefecture in the north, Goluo Tibetan Autonomous Prefecture in the east, Ganzi Tibetan Autonomous Prefecture in Sichuan Province in the southeast, and Changdu City of Tibet Autonomous Region in the south. The forest resources and animal resources are very rich. | 12700 km^2^  ~4500 m  4.6 ℃  ~540 mm | Zhaqu, Ziqu, Baqu, Requ, Jiqu | Qunguo Zhaxi Beach, Nangqian County Rancha Grand Canyon, Gaer Monastery |
| Yushu | 95°41′~97°44′ E; 33°44′~33°46′ N. Yushu City is located in the eastern part of the Tibetan Plateau, bordering to the Tibet Autonomous Region in the east and southeast, neighboring to Nangqian County in the southwest, contiguous with Zaduo County in the west, linked with Zhiduo County in the northwest, and is adjacent to Qumalai, Chengduo County and Shiqu County of Ganzi Tibetan Autonomous Prefecture of the Sichuan Province in the north and northeast; and straddling the Yangtze River and Lancang River systems, water resources and wildlife resources are also very rich. | ~15700 km^2^  ~4490 m  2.9 ℃  ~480 mm | Yangtze River, Yellow River and Lancang River source; Yangtze and Lancang River tributaries, Tongtian River, Zhaqu and Baqu | Drying Scripture Platform, Lepagou Rock Painting, Jiana Mani Stone Sutra City, Batang Hot Water Gorge, Longbaotan Nature Reserve, Batang Grassland, Ningji Lake, Zangniang Stupa, Sangchou Monastery, Dangka Monastery, Jiegu Monastery, Wencheng Princess Monastery, Changu Monastery, Banqing Monastery, Jiran Monastery |
| Chenduo | 96°02′~97°21′ E; 32°53′~34°47′ N. Chengduo County, located in the central part of Qinghai Province, bordering to Qumalai County in the north and west, adjacent to the Shiqu County of Sichuan Province in the southeast, separated by the Tongtian River to Yushu County in the southwest, there are the Yellow River water system, the Tongtian River water system, rich animal and biological resources. | 15300 km^2^  ~4500 m  -1.6 ℃  ~500 mm | Yellow River water system (Luoqu, Beiminqu), Lalang Qingqu (Zhagenjialong water, Reheyange water, Nazharen water, Nazhalongcha River), Tongtian River water system (Dequ, Zhiqu), Xiqu (Luoqie water, Shaishaizhamu water) | Gaduojuewu God Mountain, Labu Guzang Village, Jiatang Grassland, Gazang Monastery, Sehang Monastery, Labu Monastery, Saiba Monastery, Ancient rock painting group |
| Dari | 98°15′~100°33′ E; 32°36′~34°15′ N. Dari County, is situated in the southeast of Qinghai Province, at the junction of Sichuan, Gansu and Qinghai provinces, bordered to Jiuzhi County in the east, to Banma County in the southeast, adjacent to Seda County of Sichuan Province in the south, to Shiqu County of Sichuan Province in the southwest, bordered by Maduo County in the northwest, separated by the Yellow River to Maqin, Gande County in the north, is one of the “Three Rivers” natural ecological protection and management areas, including the Yangtze River, the Yellow River two water systems, animal and biological resources are abundant. | 14800 km^2^  ~4200 m  -0.5 ℃  ~595 mm | Yellow River water system, Yangtze River water system | Chalang Monastery, Shilong Palace, Gesar Linka, Zhumu Square, Golog Peaceful Liberation Monument |
| Jiuzhi | 100°20′~101°47′ E; 33°02′~34°03′ N. Jiuji County, located in the eastern part of the Qinghai-Tibet Plateau, bordered to Aba County of Sichuan Province in the southeast, Maqu County of Gansu Province in the northeast, to Dari County in the west, connected to Banma County in the south, there are many rivers and lakes in the territory, water resources, pasture resources and animal resources are very rich. | 8750 km^2^  ~4000 m  0.1 ℃  ~760 mm | Yellow River water system, Yangtze River water system (Make River, Keke River, Shake River, Haqu, Jiuqu, Zhangku River, Zhean Muku River, Xicuo Lake) | Nianbao Yuzhe, Baiyu Monastery,  Nianbao Fairy Lake, Baiyu Hot Spring, Yueya Lake, Longka Lake |
| Henan | 100°53′~102°15′ E; 34°55′~34°73′ N. Henan Mongolian Autonomous County is located in the eastern part of the Qinghai-Tibet Plateau and the southeastern part of Qinghai Province, at the southern end of the chord corresponding to the Yellow River. It borders Xiahe County and Luqu County, Maqu County, Maqin County, and Zeku County. It belongs to a typical region of slightly dissected high mountains. The county has a plateau continental climate, falling into the category of the plateau subfrigid humid climate zone. It boasted dense river network, its terrestrial vertebrate fauna alone comprises more than 100 species. | 6700 km^2^  ~3600 m  -9.2 ℃  ~600 mm | Tao River, Zequ River, Gamari River | Laka Monastery, Quge Monastery, Xiangzha Monastery, Dashen Monastery, Holy Lake, Fairy Cave, Mount Jigang, Yellow River Grand Canyon, Mount Licharu |
| Maqin | 98°00′~100°56′ E; 33°43′~35°16′ N. Maqin County, is located in the southeastern part of Qinghai Province, in the national “Three Rivers” ecological reserve, adjacent to Maqu County of Gansu Province, to Henan Mongolian Autonomous County in the northeast, to Tongde and Xinghai County, to Maduo and Dari County in the west, to Gande County in the south; forestry resources, wild animals, pasture resources, alpine meadows, alpine swamps, scrubs, mountain grasslands, alpine grasslands are abundant. | 13400 km^2^  ~4100 m  -3.9 ℃  ~490 mm | Yellow River water system (Qiemuqu River) | Animaqing Snow Mountain, Yangyu Primeval Forest, Lajia Monastery, White Pagoda, Anigetuo Sacred Mountain |
| Xinghai | 99°01′~100°59′ E; 34°48′~36°14′ N. Xinghai County, is located in the eastern part of the Qinghai-Tibetan Plateau, in the “Three Rivers” national nature protection core area, separated by the Yellow River to Guinan and Tongde County in the east, to Dulan County in the west, to Maqin and Maduo County in the south, to Gonghe County in the north; water resources, animal and biological resources are abundant. | 12100 km^2^  ~3900 m  -5 ℃  ~11 ℃  ~190 mm  ~470 mm | Yellow River water system (Qushian River, Daba River) | Yellow River Gorge, Saizong Monastery, Saizong Mountain, |

**References**

1. <https://www.yushuzhou.gov.cn/html/10/Item.html>

2. <http://www.guoluo.gov.cn/zjgl.html>

3. <http://www.huangnan.gov.cn/index.html>

4. <http://www.hainanzhou.gov.cn/>

5. <http://www.tcmap.com.cn/>

6. <https://baike.baidu.com/>
