# Supplementary material for: Assessment of environmental contamination with Echinococcus spp. through DNA detection in free-roaming canid feces and soil in human echinococcosis hotspots from the Three-River-Source Region of the Qinghai-Tibet Plateau, China
Source: Parasit Vectors. 2026 Mar 23;19:192. doi: 10.1186/s13071-026-07369-2 (PMC13130420; doi:10.1186/s13071-026-07369-2)
Supplement: Supplementary file 2 — Additional file 2: Table S1. The presence and prevalence of Echinococcus species in different hosts from different geographic origins from related literature. [file 13071_2026_7369_MOESM2_ESM.docx]

Additional file 2 Table S1 The presence and prevalence of *Echinococcus* species in different hosts from different geographic origins from related literature

| County | Year | Hosts (intermediate or definitive hosts) | Methods | *Echinococcus* species | Positive rate (No. positive/No. sampled) | Reference |
| --- | --- | --- | --- | --- | --- | --- |
| Zhiduo | 2006 | Humans | IHA | *Echinococcus* spp. | 4.50% (42/933) | (Wu et al. 2007) |
|  | 2006 | Humans | ELISA | *Echinococcus* spp. | 8.16% (76/931) | (Wu et al. 2007) |
|  | 2006 | Humans | B ultrasound | *Echinococcus* spp.  *E. granulosus*  *E. multilocularis* | 3.37% (33/979)  3.17% (31/979)  0.20% (2/979) | (Wu et al. 2007) |
|  | 2012 | Humans (Students) | B ultrasound | *Echinococcus* spp. | 0.80% (6/753) | (Cheng et al. 2018) |
|  | 2012 | Humans (Students) | ELISA | *Echinococcus* spp. | 4.65% (35/753) | (Cheng et al. 2018) |
|  | 2012 | Humans | B ultrasound | *Echinococcus* spp. | 5.35% (30/561) | (Cheng et al. 2016) |
|  | 2012 | Humans | ELISA | *Echinococcus* spp. | 11.41% (56/491) | (Cheng et al. 2016) |
|  | 2006 | Pikas | Dissection | *Echinococcus* spp.  *E. shiquicus* | 15.05% (14/93)  1.08% (1/93) | (Wu et al. 2007) |
|  | 2012 | Cattle and Sheep | Dissection | *Echinococcus* spp. | 2.32% (6/259) | (Cheng et al. 2016) |
|  | 2006 | Dogs | ELISA | *Echinococcus* spp. | 6.22% (12/193) | (Wu et al. 2007) |
|  | 2006 | Wolves | ELISA | *Echinococcus* spp. | 35.71% (5/14) | (Wu et al. 2007) |
|  | 2012 | Dogs | ELISA | *Echinococcus* spp. | 7.25% (14/193) | (Cheng et al. 2016) |
|  | 2015 | Dogs | ELISA | *E. granulosus* | 1.02% (1/98) | (Hu et al. 2015) |
| Qumalai | 2006 | Humans (Students) | B ultrasound | *Echinococcus* spp. | 1.14% (14/1228) | (Cheng et al. 2018) |
|  | 2006 | Humans (Students) | ELISA | *Echinococcus* spp. | 6.68% (82/1228) | (Cheng et al. 2018) |
|  | 2012 | Humans | B ultrasound | *Echinococcus* spp. | 0.01% (1/982) | (Cheng et al. 2016) |
|  | 2012 | Humans | ELISA | *Echinococcus* spp. | 10.29% (21/204) | (Cheng et al. 2016) |
|  | 2010 | Yaks | Dissection | *Echinococcus* spp. | 44.44% (68/153) | (Yang 2011) |
|  | 2010 | Sheep | Dissection | *Echinococcus* spp. | 54.51% (314/576) | (Yang 2011) |
|  | 2012 | Cattle and Sheep | Dissection | *Echinococcus* spp. | 1.64% (2/122) | (Cheng et al. 2016) |
|  | 2015 | Dogs | ELISA | *E. granulosus* | 13.00% (13/100) | (Hu et al. 2015) |
| Nangqian | 2012 | Humans (Students) | B ultrasound | *Echinococcus* spp. | 0.19% (3/1544) | (Cheng et al. 2018) |
|  | 2012 | Humans (Students) | ELISA | *Echinococcus* spp. | 2.20% (3/1544) | (Cheng et al. 2018) |
|  | 2012 | Humans | B ultrasound | *Echinococcus* spp. | 3.09% (32/1035) | (Cheng et al. 2016) |
|  | 2012 | Humans | ELISA | *Echinococcus* spp. | 13.07% (69/528) | (Cheng et al. 2016) |
|  | 2012 | Dogs | ELISA | *Echinococcus* spp. | 5.86% (17/290) | (Cheng et al. 2016) |
|  | 2015 | Dogs | ELISA | *E. granulosus* | 2.97% (3/101) | (Hu et al. 2015) |
| Yushu | 2003 | Humans | ELISA, B ultrasound | *E. granulosus* | 2.62% (21/801) | (He et al. 2003) |
|  | 2003 | Humans | ELISA, B ultrasound | *E. multilocularis* | 0.50% (4/801) | (He et al. 2003) |
|  | 2005 | Humans | ELISA, B ultrasound | *E. granulosus* | 2.62% (28/1070) | (Ma et al. 2005) |
|  | 2005 | Humans | ELISA, B ultrasound | *E. multilocularis* | 0.37% (4/1070) | (Ma et al. 2005) |
|  | 2008 | Humans | B ultrasound | *Echinococcus* spp. | 3.10% (25/801) | (Liu et al. 2008) |
|  | 2008 | Humans | IHA | *Echinococcus* spp. | 4.10% (33/801) | (Liu et al. 2008) |
|  | 2011 | Humans | ICS | *Echinococcus* spp. | 8.88% (15/169) | (Qiao and Duo 2014) |
|  | 2012 | Humans | B ultrasound | *Echinococcus* spp. | 2.80% (30/1070) | (Cheng et al. 2016) |
|  | 2012 | Humans | ELISA | *Echinococcus* spp. | 23.18% (127/548) | (Cheng et al. 2016) |
|  | 2014 | Humans (Children) | B ultrasound | *Echinococcus* spp. | 0.01% (1/1978) | (Xiu-Min et al. 2017) |
|  | 2014 | Humans (Children) | ELISA | *Echinococcus* spp. | 3.89% (76/1954) | (Xiu-Min et al. 2017) |
|  | 2008 | Sheep | Dissection | *Echinococcus* spp. | 25.10% (64/255) | (Pengmao 2009) |
|  | 2008 | Yaks | Dissection | *Echinococcus* spp. | 25.60% (33/129) | (Pengmao 2010) |
|  | 2012 | Cattle and Sheep | Dissection | *Echinococcus* spp. | 20.61% (74/359) | (Cheng et al. 2016) |
|  | 2018 | Pikas | PCR | *E. shiquicus* | 2.10% (3/143) | (Wang et al. 2021) |
|  | 2011 | Dogs | PCR | *Echinococcus* spp. | 27.66% (26/94) | (Dang et al. 2017) |
|  | 2015 | Dogs | ELISA | *E. granulosus* | 9.49% (13/137) | (Hu et al. 2015) |
|  | 2015 | Dogs | IHA | *Echinococcus* spp. | 10.00% (9/90) | (Wei et al. 2020) |
|  | 2016 | Dogs | IHA | *Echinococcus* spp. | 6.67% (8/120) |  |
|  | 2017 | Dogs | IHA | *Echinococcus* spp. | 4.44% (8/180) |  |
|  | 2018 | Dogs | IHA | *Echinococcus* spp. | 3.89% (7/180) |  |
|  | 2019 | Dogs | IHA | *Echinococcus* spp. | 2.78% (5/180) |  |
|  | 2020 | Foxex | PCR | *Echinococcus* spp. | 7.89% (22/279) | (Zhang et al. 2022) |
| Chenduo | 2008 | Humans | B ultrasound | *Echinococcus* spp.  *E. granulosus*  *E. multilocularis* | 13.04% (6/46)  4.35% (2/46)  8.70% (4/46) | (Fu et al. 2010) |
|  | 2008 | Humans | B ultrasound | *Echinococcus* spp. | 5.99% (48/801) | (Liu et al. 2008) |
|  | 2008 | Humans | IHA | *Echinococcus* spp. | 25.48% (132/518) | (Liu et al. 2008) |
|  | 2014 | Humans (Children) | B ultrasound | *Echinococcus* spp. | 1.11% (18/1612) | (Han et al. 2017) |
|  | 2014 | Humans (Children) | ELISA | *Echinococcus* spp. | 4.68% (75/1602) | (Han et al. 2017) |
|  | 2014 | Humans | B ultrasound | *Echinococcus* spp.  *E. granulosus*  *E. multilocularis* | 7.84% (271/3457)  2.20% (76/3457)  5.64% (195/3457) | (Ding 2016) |
|  | 2008 | Pikas | Dissection | *E. multilocularis* | 4.36% (23/527) | (Fu et al. 2010) |
|  | 2008 | Voles | Dissection | *E. multilocularis* | 1.00% (4/409) | (Fu et al. 2010) |
|  | 2008 | Dogs | ELISA | *Echinococcus* spp. | 32.58% (43/132) | (Fu et al. 2010) |
|  | 2010 | Dogs | PCR | *E. granulosus* | 8.33% (6/72) | (Guo et al. 2014) |
|  | 2011 | Dogs | PCR | *Echinococcus* spp. | 44.19% (38/86) | (Dang et al. 2017) |
|  | 2012 | Dogs | PCR | *E. multilocularis* | 33.33% (2/6) Case | (Feng et al. 2013) |
|  | 2015 | Dogs | ELISA | *E. granulosus* | 19.59% (19/97) | (Hu et al. 2015) |
|  | 2011 | Foxex | PCR | *Echinococcus* spp. | 66.67% (4/6) | (Dang et al. 2017) |
|  | 2012 | Foxex | PCR | *E. multilocularis* | 33.33% (2/6) Case | (Feng et al. 2013) |
|  | 2012 | Foxex | PCR | *E. shiquicus* | 16.67% (1/6) Case | (Feng et al. 2013) |
| Jiuzhi | 2005 | Humans | B ultrasound | *Echinococcus* spp.  *E. granulosus*  *E. multilocularis* | 8.00% (124/1549)  5.49% (85/1549)  2.52% (39/1549) | (Yu et al. 2008) |
|  | 2005 | Humans | IHA | *Echinococcus* spp. | 25.79% (287/1113) | (Yu et al. 2008) |
|  | 2006 | Humans | B ultrasound | *Echinococcus* spp.  *E. granulosus*  *E. multilocularis* | 8.01% (124/1549)  5.42% (84/1549)  2.52% (39/1549) | (Wu et al. 2007) |
|  | 2011 | Humans (Children) | B ultrasound | *Echinococcus* spp.  *E. granulosus*  *E. multilocularis* | 1.58% (30/1901)  0.95% (18/1901)  0.63% (12/1901) | (Cai et al. 2017) |
|  | 2012 | Humans | ELISA | *Echinococcus* spp. | 1.05% (34/3226) | (Ma et al. 2017) |
|  | 2014 | Humans (Children) | B ultrasound | *Echinococcus* spp. | 1.57% (30/1909) | (Gongque 2015) |
|  | 2014 | Humans (Children) | ELISA | *Echinococcus* spp. | 19.94% (255/1279) | (Gongque 2015) |
|  | 2014 | Humans (Children) | B ultrasound | *Echinococcus* spp. | 0.63% (12/1908) | (Han et al. 2017) |
|  | 2014 | Humans (Children) | ELISA | *Echinococcus* spp. | 19.95% (255/1278) | (Han et al. 2017) |
|  | 2017 | Humans | ELISA, B ultrasound | *E. multilocularis* | 80 Case | (Wang et al. 2022) |
|  | 2017 | Humans | B ultrasound | *Echinococcus* spp.  *E. granulosus*  *E. multilocularis* | 3.16% (798/25238)  0.97% (245/25238)  2.18% (549/25238) | (Liu et al. 2021) |
|  | 2018 | Humans | B ultrasound | *Echinococcus* spp.  *E. granulosus*  *E. multilocularis* | 1.47% (345/23505)  0.54% (126/23505)  0.93% (219/23505) | (Gao et al. 2019) |
|  | 2001 | Yaks | Dissection | *Echinococcus* spp. | 57.23% (198/346) | (Danba 2003) |
|  | 2005 | Yaks | Dissection | *E. granulosus* | 78.52% (106/135) | (Yu et al. 2008) |
|  | 2006 | Yaks | Dissection | *Echinococcus* spp. | 32.43% (168/518) | (Zhou and Yang 2009) |
|  | 2007 | Yaks | Dissection | *Echinococcus* spp. | 32.04% (165/515) | (Hu and Angqing 2008) |
|  | 2012 | Yaks | Dissection | *Echinococcus* spp. | 9.60% (48/500) | (Ma et al. 2017) |
|  | 2005 | Sheep | Dissection | *E. granulosus* | 82.61% (95/115) | (Yu et al. 2008) |
|  | 2017 | Sheep | Dissection | *Echinococcus* spp. | 35.22% (81/230) | (He 2019) |
|  | 2017 | Sheep | Dissection | *Echinococcus* spp. | 78.67% (118/150) | (Caiji 2019) |
|  | 2012 | Rodents | Dissection | *Echinococcus* spp. | 4.82% (49/1016) | (Ma et al. 2017) |
|  | 2017 | Pikas | Dissection, PCR | *E. multilocularis* | 4.08% (2/49) | (Li et al. 2018) |
|  | 2017 | Voles | Dissection, PCR | *E. multilocularis* | 29.41% (30/102) | (Li et al. 2018) |
|  | 2005 | Dogs | Dissection | *Echinococcus* spp.  *E. granulosus*  *E. multilocularis* | 75.00% (9/12) Case  66.67% (8/12)  8.33% (1/12) | (Yu et al. 2008) |
|  | 2005 | Dogs | PCR | *E. granulosus* | 4.70% (7/149) | (Yu et al. 2008) |
|  | 2012 | Dogs | ELISA | *Echinococcus* spp. | 14.38% (46/320) | (Ma et al. 2017) |
|  | 2021 | Dogs | Dissection, ELISA | *Echinococcus* spp. | 9.78% (9/92) | (Geng et al. 2022) |
| Dari | 2007 | Humans | B ultrasound | *Echinococcus* spp.  *E. granulosus*  *E. multilocularis* | 13.70% (236/1723)  5.51% (95/1723)  8.18% (141/1723) | (Han et al. 2009) |
|  | 2007 | Humans | ELISA | *Echinococcus* spp. | 21.69% (252/1162) | (Han et al. 2009) |
|  | 2010 | Humans | B ultrasound | *Echinococcus* spp. | 10.16% (387/3810) | (Wei 2011) |
|  | 2010 | Humans | ELISA | *Echinococcus* spp. | 15.57% (427/2742) | (Wei 2011) |
|  | 2011 | Humans (Children) | B ultrasound | *Echinococcus* spp.  *E. granulosus*  *E. multilocularis* | 4.08% (100/2449)  0.82% (20/2449)  3.27% (80/2449) | (Cai et al. 2017) |
|  | 2012 | Humans | B ultrasound | *Echinococcus* spp. | 11.93% (430/3605) | (Ma et al. 2017) |
|  | 2014 | Humans (Children) | B ultrasound | *Echinococcus* spp. | 4.31% (106/2461) | (Han et al. 2017) |
|  | 2014 | Humans (Children) | ELISA | *Echinococcus* spp. | 23.02% (429/1864) | (Han et al. 2017) |
|  | 2007 | Yaks | Dissection, PCR | *E. granulosus* | 26.42% (14/53) | (Han et al. 2009) |
|  | 2012 | Yaks | Dissection | *Echinococcus* spp. | 62.80% (314/500) | (Ma et al. 2017) |
|  | 2007 | Sheep | Dissection, PCR | *E. granulosus* | 31.25% (5/16) | (Han et al. 2009) |
|  | 2007 | Pikas | Dissection, PCR | *E. shiquicus* | 11.30% (27/239) | (Han et al. 2009) |
|  | 2012 | Rodents | Dissection | *Echinococcus* spp. | 2.88% (29/1006) | (Ma et al. 2017) |
|  | 2007 | Dogs | Dissection | *Echinococcus* spp.  *E. granulosus*  *E. multilocularis* | 55.56% (5/9) Case  11.11% (1/9) Case  44.44% (4/9) Case | (Han et al. 2009) |
|  | 2007 | Dogs | ELISA | *Echinococcus* spp. | 11.27% (31/275) | (Han et al. 2009) |
|  | 2012 | Dogs | ELISA | *Echinococcus* spp. | 9.06% (29/320) | (Ma et al. 2017) |
|  | 2021 | Dogs | Dissection, ELISA | *Echinococcus* spp. | 2.22% (8/360) | (Geng et al. 2022) |
| Zaduo | 2012 | Humans | B ultrasound | *Echinococcus* spp. | 4.82% (45/933) | (Cheng et al. 2016) |
|  | 2012 | Humans | ELISA | *Echinococcus* spp. | 22.27% (147/660) | (Cheng et al. 2016) |
|  | 2012 | Dogs | ELISA | *Echinococcus* spp. | 10.68% (11/103) | (Cheng et al. 2016) |
|  | 2015 | Dogs | ELISA | *E. granulosus* | 5.00% (5/100) | (Hu et al. 2015) |
| Zeku | 2000 | Humans | B ultrasound | *Echinococcus* spp.  *E. granulosus*  *E. multilocularis* | 7.65% (80/1046)  7.36% (77/1046)  0.29% (3/1046) | (He and Wang 2001) |
|  | 2010 | Humans | B ultrasound | *Echinococcus* spp.  *E. granulosus*  *E. multilocularis* | 2.94% (87/2959)  2.94% (87/2959)  0.03% (1/2959) | (Chen 2013) |
|  | 2000 | Yaks | Dissection | *Echinococcus* spp.  *E. granulosus*  *E. multilocularis* | 84.60% (324/383)  79.90% (306/383)  4.70% (18/383) | (He and Wang 2001) |
|  | 2000 | Sheep | Dissection | *Echinococcus* spp.  *E. granulosus*  *E. multilocularis* | 89.97% (520/578)  84.60% (489/578)  5.36% (31/578) | (He and Wang 2001) |
|  | 2000 | Pikas | Dissection | *E. multilocularis* | 3.45% (11/319) | (He and Wang 2001) |
|  | 2010 | Pikas | Dissection | *Echinococcus* spp. | 0.42% (2/472) | (Chen 2013) |
|  | 2000 | Voles | Dissection | *E. multilocularis* | 12.50% (1/8) | (He and Wang 2001) |
|  | 2000 | Dogs | Dissection | *E. granulosus*  *E. multilocularis* | 41.67% (5/12)  8.33% (1/12) | (He and Wang 2001) |
|  | 2010 | Dogs | ELISA | *Echinococcus* spp. | 13.33% (20/150) | (Chen 2013) |
| Maqin | 2008 | Humans | B ultrasound | *Echinococcus* spp.  *E. granulosus*  *E. multilocularis* | 7.43% (116/1561)  5.25% (82/1561)  2.18% (34/1561) | (Ma et al. 2015) |
|  | 2008 | Humans | ELISA | *Echinococcus* spp. | 23.84% (307/1288) | (Ma et al. 2015) |
|  | 2011 | Humans (Children) | B ultrasound | *Echinococcus* spp.  *E. granulosus*  *E. multilocularis* | 1.12% (35/3115)  0.55% (17/3115)  0.58% (18/3115) | (Cai et al. 2017) |
|  | 2012 | Humans | B ultrasound | *Echinococcus* spp. | 0.56% (18/3202) | (Ma et al. 2017) |
|  | 2014 | Humans (Children) | B ultrasound | *Echinococcus* spp. | 0.67% (21/3135) | (Han et al. 2017) |
|  | 2014 | Humans (Children) | ELISA | *Echinococcus* spp. | 20.61% (498/2416) | (Han et al. 2017) |
|  | 2018 | Humans | B ultrasound | *Echinococcus* spp. | 1.18% (433/36705) | (Ma et al. 2020) |
|  | 2005 | Yaks | Dissection | *Echinococcus* spp. | 8.16% (40/490) | (Cuomaoji 2006) |
|  | 2012 | Yaks | Dissection | *Echinococcus* spp. | 5.20% (26/500) | (Ma et al. 2017) |
|  | 2017 | Yaks | Dissection | *Echinococcus* spp. | 8.27% (40/490) | (Zhoumaocuo 2018) |
|  | 2005 | Sheep | Dissection | *Echinococcus* spp. | 61.40% (692/1127) | (Cuomaoji 2006) |
|  | 2017 | Sheep | Dissection | *Echinococcus* spp. | 62.21% (701/1127) | (Zhoumaocuo 2018) |
|  | 2012 | Rodents | Dissection | *Echinococcus* spp. | 0.29% (3/1036) | (Ma et al. 2017) |
|  | 2008 | Dogs | ELISA | *Echinococcus* spp. | 27.14% (54/199) | (Ma et al. 2015) |
|  | 2012 | Dogs | ELISA | *Echinococcus* spp. | 18.76% (160/853) | (Ma et al. 2017) |
|  | 2019 | Dogs | ELISA | *Echinococcus* spp. | 13.11% (8/61) | (Wei et al. 2020) |
|  | 2019 | Dogs | PCR | *Echinococcus* spp.  *E. granulosus*  *E. multilocularis* | 25.00% (36/144)  25.00% (36/144)  0.69% (1/144) | (Cui et al. 2020) |
|  | 2021 | Dogs | Dissection, ELISA | *Echinococcus* spp. | 5.28% (19/360) | (Geng et al. 2022) |
|  | 2019 | Foxes | ELISA | *Echinococcus* spp. | 3.73% (6/161) | (Wei et al. 2020) |
|  | 2019 | Wolves | ELISA | *Echinococcus* spp. | 6.67% (2/30) | (Wei et al. 2020) |
| Xinghai | 2001 | Humans | B ultrasound | *Echinococcus* spp.  *E. granulosus*  *E. multilocularis* | 1.44% (9/627)  1.28% (8/627)  0.16% (1/627) | (Wu et al. 2001) |
|  | 2001 | Humans | ID and IHA | *Echinococcus* spp. | 6.38% (40/627) | (Wu et al. 2001) |
|  | 2009  2010  2011  2012  2013  2014  2015 | Humans (Children)  Humans (Children)  Humans (Children)  Humans (Children)  Humans (Children)  Humans (Children)  Humans (Children) | B ultrasound  B ultrasound  B ultrasound  B ultrasound  B ultrasound  B ultrasound  B ultrasound | *Echinococcus* spp.  *Echinococcus* spp.  *Echinococcus* spp.  *Echinococcus* spp.  *Echinococcus* spp.  *Echinococcus* spp.  *Echinococcus* spp. | 0.16% (1/608)  0.18% (2/1140)  0.06% (2/3297)  0.21% (2/960)  0.25% (3/1216)  0.00% (0/383)  0.17% (2/1174) | (Xiao 2016) |
|  | 2009  2010  2011  2012  2013  2014  2015 | Humans (Children)  Humans (Children)  Humans (Children)  Humans (Children)  Humans (Children)  Humans (Children)  Humans (Children) | ELISA  ELISA  ELISA  ELISA  ELISA  ELISA  ELISA | *Echinococcus* spp.  *Echinococcus* spp.  *Echinococcus* spp.  *Echinococcus* spp.  *Echinococcus* spp.  *Echinococcus* spp.  *Echinococcus* spp. | 10.87% (66/607)  10.53% (40/380)  4.60% (46/999)  11.54% (39/338)  0.74% (9/1216)  0.00% (0/383)  2.30% (27/1174) | (Xiao 2016) |
|  | 2015 | Humans | B ultrasound, ELISA | *E. granulosus* | 0.16% (5/3223) | (Cai et al. 2016) |
|  | 2008 | Yaks | Dissection | *Echinococcus* spp. | 48.70% (306/629) | (Gengtaiyou 2009) |
|  | 2009  2010  2011  2012  2013  2014  2015 | Yaks  Sheep  Sheep  Sheep  Yaks  Sheep  Yaks, Sheep | Dissection  Dissection  Dissection  Dissection  Dissection  Dissection  Dissection | *Echinococcus* spp.  *Echinococcus* spp.  *Echinococcus* spp.  *Echinococcus* spp.  *Echinococcus* spp.  *Echinococcus* spp.  *Echinococcus* spp. | 5.48% (17/310)  2.80% (5/180)  2.70% (4/150)  5.37% (27/503)  3.00% (3/100)  0.40% (2/500)  2.31% (7/303) | (Xiao 2016) |
|  | 2014 | Yaks | Dissection | *Echinococcus* spp. | 7.14% (40/560) | (Yuan 2015) |
|  | 2014 | Sheep | Dissection | *Echinococcus* spp. | 20.00% (130/650) | (Yuan 2015) |
|  | 2001 | Dogs | Deworming, Observation | *Echinococcus* spp. | 12.50% (1/8) | (Wu et al. 2001) |
|  | 2010 | Dogs | PCR | *E. granulosus* | 13.33% (12/90) | (Guo et al. 2014) |
|  | 2009  2010  2011  2012  2013  2014  2015 | Dogs  Dogs  Dogs  Dogs  Dogs  Dogs  Dogs | Dissection, ELISA  Dissection, ELISA  Dissection, ELISA  Dissection, ELISA  Dissection, ELISA  Dissection, ELISA  Dissection, ELISA | *Echinococcus* spp.  *Echinococcus* spp.  *Echinococcus* spp.  *Echinococcus* spp.  *Echinococcus* spp.  *Echinococcus* spp.  *Echinococcus* spp. | 6.17% (35/567)  2.61% (45/1723)  4.16% (20/481)  13.75% (44/320)  2.00% (31/1547)  5.60% (93/1662)  7.00% (7/100) | (Xiao 2016) |
| Banma | 2011 | Humans (Children) | B ultrasound | *Echinococcus* spp.  *E. granulosus*  *E. multilocularis* | 1.93% (26/1349)  0.22% (3/1349)  1.70% (23/1349) | (Cai et al. 2017) |
| Banma | 2014 | Humans (Children) | B ultrasound | *Echinococcus* spp. | 1.92% (26/1351) | (Han et al. 2017) |
| Banma | 2014 | Humans (Children) | ELISA | *Echinococcus* spp. | 14.09% (167/1185) | (Han et al. 2017) |
| Banma | 2021 | Dogs | Dissection, ELISA | *Echinococcus* spp. | 1.11% (3/270) | (Geng et al. 2022) |
| Banma | 2012 | Humans | B ultrasound | *Echinococcus* spp. | 6.12% (151/2466) | (Ma et al. 2017) |
| Banma | 2012 | Humans | ELISA | *Echinococcus* spp. | 12.77% (136/1065) | (Ma et al. 2017) |
| Gande | 2014 | Humans (Children) | B ultrasound | *Echinococcus* spp. | 0.78% (19/2449) | (Han et al. 2017) |
| Gande | 2014 | Humans (Children) | ELISA | *Echinococcus* spp. | 19.96% (454/2274) | (Han et al. 2017) |
| Gande | 2011 | Humans (Children) | B ultrasound | *Echinococcus* spp.  *E. granulosus*  *E. multilocularis* | 1.80% (44/2446)  1.27% (31/2446)  0.53% (13/2446) | (Cai et al. 2017) |
| Gande | 2021 | Dogs | Dissection, ELISA | *Echinococcus* spp. | 1.46% (4/274) | (Geng et al. 2022) |
| Gande | 2012 | Humans | B ultrasound | *Echinococcus* spp. | 5.77% (188/3256) | (Ma et al. 2017) |
| Gande | 2012 | Humans | ELISA | *Echinococcus* spp. | 48.15% (65/135) | (Ma et al. 2017) |
| Gande | 2012 | Cattle | Dissection | *Echinococcus* spp. | 35.76% (182/509) | (Ma et al. 2017) |
| Gande | 2012 | Rodents | Dissection | *Echinococcus* spp. | 1.70% (17/1000) | (Ma et al. 2017) |
| Gande | 2012 | Dogs | ELISA | *Echinococcus* spp. | 14.38% (46/320) | (Ma et al. 2017) |
| Maduo | 2012 | Humans | B ultrasound | *Echinococcus* spp. | 3.70% (5/135) | (Ma et al. 2017) |
| Maduo | 2014 | Humans (Children) | B ultrasound | *Echinococcus* spp. | 0.69% (6/871) | (Han et al. 2017) |
| Maduo | 2014 | Humans (Children) | ELISA | *Echinococcus* spp. | 3.67% (32/871) | (Han et al. 2017) |
| Maduo | 2012 | Dogs | ELISA | *Echinococcus* spp. | 18.76% (160/853) | (Ma et al. 2017) |
| Maduo | 2021 | Dogs | Dissection, ELISA | *Echinococcus* spp. | 3.30% (6/182) | (Geng et al. 2022) |

Note:

IHA: indirect hemagglutination assay.

ELISA: Enzyme-linked immunosorbent assay.

B ultrasound: Type B ultrasonic examination.

Dissection: Examination of the surface of the livers and lungs of intermediate hosts after slaughter; collection of intestinal contents of the definitive hosts after slaughter, observation examination under the microscope.

ICS: immunochromatographic strip.

PCR: Polymerase chain reaction.

ID: Casoni’s intradermal test.

Deworming: Oral deworming to the definitive hosts.

**References**

Cai, H., Guan, Y., Ma, X., Wang, L., Wang, H., Su, G., Zhang, X., Han, X., Ma, J., Liu, Y.F., Li, J., Zhang, J., Wang, Y., Wang, W., Du, R., Lei, W. and Wu, W. (2017) Epidemiology of Echinococcosis Among Schoolchildren in Golog Tibetan Autonomous Prefecture, Qinghai, China. Am J Trop Med Hyg 96(3), 674-679.

Cheng, S.L., Wang, H., Ma, X., Zhang, J.X., Liu, Y.F., Cai, H.X., Liu, P.Y., Ma, J.Y., He, D.L., Wu, X.H., Han, X.M., Wang, Y.S., Liu, H.Q., Zhao, Y.M., Liu, B.R., Zeng, C., Wang, W., Du, R., Lei, W., Su, G.M., Zhou, B.J., Song, C.X. and Wang, Y.J. (2016) [An Epidemiological Survey on Echinococcosis in Yushu Prefecture of Qinghai Province]. Zhongguo Ji Sheng Chong Xue Yu Ji Sheng Chong Bing Za Zhi 34(6), 547-551.

Dang, Z., Fu, Y., Duo, H., Fan, H., Qiao, Z., Guo, Z., Feng, K., Chui, W., Shen, X., Geng Qiu, J., Ni, M., He, S., Zhao, H., Peng, M., Xiao, N., Nonaka, N., Nasu, T., Huang, F., Oku, Y., Hayashimoto, N., Hu, W. and Li, W. (2017) An epidemiological survey of echinococcosis in intermediate and definitive hosts in Qinghai Province, China. Trop Biomed 34(2), 483-490.

Feng, K., Huang, F.Q., Duo, H., Fu, Y., Shen, X.Y., Peng, M. and Li, W. (2013) [Species identification of Echinococcus isolates collected from canines and Tibetan foxes in Chengduo County, Qinghai Province]. Zhongguo Ji Sheng Chong Xue Yu Ji Sheng Chong Bing Za Zhi 31(3), 185-187.

Fu, Q., Han, X.M., Wang, L.Y., Sangba, D.Y., Ma, X., Wang, Y.S. and Wu, W.P. (2010) [Investigation on epidemic status of echinococcosis in pastoral villages of Chengduo county, Qinghai]. Zhonghua Liu Xing Bing Xue Za Zhi 31(4), 471-472.

Guo, Z., Li, W., Peng, M., Duo, H., Shen, X., Fu, Y., Irie, T., Gan, T., Kirino, Y., Nasu, T., Horii, Y. and Nonaka, N. (2014) Epidemiological study and control trial of taeniid cestode infection in farm dogs in Qinghai Province, China. J Vet Med Sci 76(3), 395-400.

Li, J.Q., Li, L., Fan, Y.L., Fu, B.Q., Zhu, X.Q., Yan, H.B. and Jia, W.Z. (2018) Genetic Diversity in Echinococcus multilocularis From the Plateau Vole and Plateau Pika in Jiuzhi County, Qinghai Province, China. Front Microbiol 9, 2632.

Wang, X., Zuo, Q.Q., Yu, Q., Song, C.X., Wang, Z.H., Xiao, N., Wang, Y.J., Weng, X.D., Wei, X., Zhou, H.R. and Cui, X.Y. (2021) Investigation on population dynamics and Echinococcus infections in small rodents around human settlement in Yushu City, Qinghai Province. Zhongguo Xue Xi Chong Bing Fang Zhi Za Zhi 33(4), 346-352. (In Chinese)

Wang, Y.X., Liu, W., Sun, Z.Y., Wu, L., Xie, X.K. and Liu, B. (2022) Analysis of Ultrasonographic Characteristics of Early Hepatic Alveolar Echinococcosis. Front Surg 9, 918138.

Wu, X.H., Wang, H., Zhang, J.X., Ma, X., Liu, Y.F., Han, X.M., Liu, H.Q., Cai, H.X., Zhao, Y.M., Ma, J.Y., Liu, P.Y. and Zeng, C. (2007) An epidemiological survey on echinococcosis in Zhiduo County of Qinghai Province. Zhongguo Ji Sheng Chong Xue Yu Ji Sheng Chong Bing Za Zhi 25(3), 229-231. (In Chinese)

Xiu-Min, H., Xue-Yong, Z., Qi-Gang, C., Jing-Ni, Z., Yong-Shun, W. and Qiang, Z. (2017) Epidemic status of alveolar echinococcosis in Tibetan children in south Qinghai Province. Zhongguo Xue Xi Chong Bing Fang Zhi Za Zhi 29(1), 53-58. (In Chinese)

Yu, S.H., Wang, H., Wu, X.H., Ma, X., Liu, P.Y., Liu, Y.F., Zhao, Y.M., Morishima, Y. and Kawanaka, M. (2008) Cystic and alveolar echinococcosis: an epidemiological survey in a Tibetan population in southeast Qinghai, China. Jpn J Infect Dis 61(3), 242-246.

Zhang, X., Fu, Y., Ma, Y., Guo, Z., Shen, X., Li, Z., Jiunai, G., Wang, X., Maji, W. and Duo, H. (2022) Brief report prevalence of Echinococcus species in wild foxes in parts of Qinghai Province, China. Vet Res Commun.

Cai, J. (2019) Investigation and prevention of echinococcosis in sheep in Jiuzhi County, Qinghai Province. Graziery Veterinary Sciences (Electronic Version), 52(16), 41-42. (In Chinese)

Cai, H. X., Wang, H., Han, X. M., Ma, X., Zhang, J. X., Liu, Y. F., Wang, Y. S., Lei, W., Wang, W., Liu, P. Y., Ma, J. Y., Su, G. M., & Du, R. (2016). A survey on echinococcosis in Hainan Tibetan Autonomous Prefecture of Qinghai Province. Journal of Pathogen Biology, 11(11), 1022-1025. (In Chinese)

Chen, Z.C. (2013) Epidemiological survey report on echinococcosis in Zeku County, Huangnan Prefecture. China Health Care & Nutrition, 23(06), 1492. (In Chinese)

Cheng, S. L., Ma, X., Cai, H. X., Liu, N., Liu, Y. F., & Shi, K. M. (2018). Epidemiological investigation of hydatid disease prevalence among students in Yushu Prefecture of Qinghai Province. Chinese Journal of Parasitology and Parasitic Diseases, 36(03), 263-265. (In Chinese)

Cui, X. Y., Gong, W. C., Han, S., Xue, C. Z., Wang, X., Ma, X., Cai, H. X., Liu, B. X., Wei, S. H., Fu, M. H., Wu, W. P., & Guan, Y. Y. (2020). The state of and risk factors for an Echinococcus infection in domestic dogs in Maqin County, Qinghai Province. Journal of Pathogen Biology, 15(06), 692-697. (In Chinese)

Cuo, M.J. (2006) Investigation on echinococcosis in cattle and sheep in Maqin County. Qinghai Journal of Animal and Veterinary Sciences, 03, 31. (In Chinese)

Dan, B. (2003) Investigation on echinococcosis in cattle in Jiuzhi County. Chinese Journal of Veterinary Medicine, 05, 33. (In Chinese)

Ding, T.L. (2016) Epidemiological analysis of echinococcosis in Chengduo County, Yushu Prefecture, Qinghai Province. Master Thesis, Qinghai University. (In Chinese)

Gao, W., Wang, H., Ma, Y.L., Zhao, S.Y., Ajide and Yang, J.Y. (2019) Epidemiological investigation of hepatic echinococcosis in human population in Jiuzhi, Qinghai Province in 2018. Chinese Journal of Parasitology and Parasitic Diseases 37(06), 681-684. (In Chinese)

Geng, P.C., Cai, J.S., Li, J. and Zhao, Q.B. (2022) Investigation on Echinococcus infection in dogs in Guoluo Prefecture, Qinghai Province. Northern Animal Husbandry, 655(15), 22-23. (In Chinese)

Gengtaiyou (2009) Investigation on echinococcosis infection in yaks in Xinghai County. Chinese Journal of Zoonoses, 25(11), 1128. (In Chinese)

Gongqueqiuge (2015) Investigation and analysis of echinococcosis among students in Jiuzhi County, Guoluo Prefecture, Qinghai Province. World Latest Medicine Information, 15(25), 160. (In Chinese)

Han, X.M., Wang, H., Cai, H.X., Ma, X., Liu, Y.F., Wei, B.H., A, I. and PS, C. (2009) Epidemiological Survey on Echinococcosis in Darlag County of Qinghai Province. Chinese Journal of Parasitology and Parasitic Diseases, 27(01), 22-26. (In Chinese)

He, D.L. and Wang, H. (2001) A Report on the Epidemiological Evaluation of Hydatid Disease in Zeku County, Qinghai Province. Endemic Diseases Bulletin (04), 36-38.. (In Chinese)

He, D.L., Wu, X.H., Liu, B.R., Liu, H.Q., Ma, X., Liu, P.Y., Liu, Y.F., Tang, X.Y. and Wang, H. (2003) An Epidemiological survey on hydatid diseases in Yushu county, Qinghai Province. Journal of Tropical Diseases and Parasitology (01), 24-25+44. (In Chinese)

He, W.J. (2019) Investigation on Echinococcus infection in slaughtered sheep in Jiuzhi County, Qinghai Province. Animal Husbandry and Veterinary Medicine Science (Electronic Edition) No.53(17), 12-13.. (In Chinese)

Hu, G.W., Li, J., Zhao, Q.B., Li, L.F., Ma, Z.Q., Cai, J.S. and Ma, R.L. (2015) Investigation on Echinococcus granulosus infection in dogs in Yushu area by fecal antigen detection using ELISA, p. 1, Harbin, Heilongjiang, China. (In Chinese)

Hu, Q.P. and Angqingzhuoma (2008) Investigation on Echinococcus infection in yaks in Baiyu Township, Jiuzhi County. Qinghai Journal of Animal and Veterinary Sciences, 197(05), 17. (In Chinese)

Liu, H.Q., He, D.L., Wu, X.H., Wang, H., Ma, X. and Zhao, Y.M. (2008) An epidemiological survey on hydatid disease in Yushu Prefecture of Qinghai Province. Chinese Journal of Parasitology and Parasitic Diseases, 26(06), 480-481+484. (In Chinese)

Liu, J., Lei, W., Ma, X., Zhang, X.F., Fan, H.N., Wang, W., Zhang, Q., Ma, J.Y., Cai, H.X., Zhan, P.Z., Zhang, J.X., Liu, Y.F., Liu, N., Wang, Y.S., Zhao, C.Z., Zhang, X.Y., Shi, K.M., Yin, X.Q. and Shang, S.X. (2021) Echinococcosis prevalence and knowledge awareness in population in Jiuzhi County of Qinghai Province during 2017—2018. Chinese Journal of Parasitology and Parasitic Diseases, 39(05), 681-686. (In Chinese)

Ma, J.Y., Ma, X., Wu, X.H., Zhang, J.X., He, D.L., Liu, Y.F., Liu, B.R., Liu, P.Y., Liu, H.Q., Zhao, Y.M., Cai, H.X. and Wang, H. (2005) Epidemiological survey on human echinococcosis in Yushu County. Chinese Journal of Parasitology and Parasitic Diseases (06), 452. (In Chinese)

Ma, X., Wang, H., Han, X.M., Zhang, J.X., Liu, Y.F., Zhao, Y.M., Wang, Y.S., Ma, J.Y., Liu, H.Q. and Gangjian (2015) Survey on Echinococcosis in Maqing County of Qinghai Province. Chinese Journal of Parasitology and Parasitic Diseases, 33(04), 269-272. (In Chinese)

Ma, X., Wang, H., Zhang, J.X., Wang, Y.S., Cheng, S.L., Liu, Y.F., Ma, J.Y., Cai, H.X., Liu, P.Y., Han, X.M., Liu, H.Q., Zhao, Y.M., Wang, W., Du, R., Lei, W., Su, G.M., Zhao, C.Z., Gangjian, Gangzhou and Qiuge (2017) Epidemiological investigation on hydatid disease/echinococcosis in Guoluo Tibetan Autonomous Prefecture in Qinghai Province. Chinese Journal of Parasitology and Parasitic Diseases, 35(04), 366-370. (In Chinese)

Ma, X., Zhang, X.F., Lei, W., Ma, J.Y., Liu, J., Cai, H.X., Wang, W., Zhan, P.Z., Fan, H.N., Zhang, J.X., Liu, Y.F., Liu, N., Wang, Y.S., Zhang, Q., Zhao, C.Z., Zhang, X.Y., Shi, K.M., Liu, P.Y., Dancuo and Changmei (2020) A survey on the prevalence and knowledge awareness of human echinococcosis in residents in Maqin County of Qinghai Province in 2017-2018. Chinese Journal of Parasitology and Parasitic Diseases, 38(05), 619-624. (In Chinese)

Peng, M. (2009) Investigation on Echinococcus infection in grazing sheep in Yushu County. China Animal Health Inspection, 26(12), 43. (In Chinese)

Peng, M. (2010) Investigation on Echinococcus infection in yaks in Yushu, Qinghai. Chinese Journal of Veterinary Medicine, 46(01), 46. (In Chinese)

Qiao, H.S. and Duo, H. (2014) Investigation and Social Factor Analysis of Echinococcosis Prevalence in Elderly Population in Yushu County Qinghai Province. Progress in Veterinary Medicine, 35(12), 182-184. (In Chinese)

Wei, B.H. (2011) Epidemiological survey and analysis of echinococcosis in Dari County, Qinghai Province. Journal of Medical Pest Control, 27(08), 746-747. (In Chinese)

Wei, S.H., Wu, W.P., Han, S., Xue, C.Z., Liu, B.X., Wang, X., Gong, W.C., Cui, X.Y. and Fu, M.H. (2020) Echinococcus eggs in canine feces in wild areas of Maqin County,Qinghai Province. Journal of Pathogen Biology, 15(05), 568-574+579. (In Chinese)

Wei, T.H., Cairen taci and Zhaxipengcuo (2020) Analysis of monitoring results of hydatid antigen in dog dung in Yushu. Chinese Qinghai Journal of Animal and Veterinary Sciences, 50(03), 44-45. (In Chinese)

Wu, X.H., Ma, X., Ning, G., He, D.L., Mao, J.S. and Wen, X. (2001) Epidemiologic survey and studies on echinococcosis in humans in Jiuzhi county of Qinghai province. Chinese Journal of Zoonoses, 23(08), 813-815. (In Chinese)

Wu, X.H., Wang, H., Nakamura, M., Morishima, Y., Ma, X., Liu, P.Y., Liu, Y.F., Zhao, Y.M., Zhang, J.X. and Yu, S.H. (2007) Epidemiologic survey and studies on echinococcosis in humans in Jiuzhi county of Qinghai province. Chinese Journal of Zoonoses, 23(08), 813-815. (In Chinese)

Xiao, C.Q. (2016) Investigation on echinococcosis infection in Xinghai County of Qinghai Province. Journal of Traditional Chinese Veterinary Medicine, 35(04), 44-46. (In Chinese)

Yang, G.X. (2011) Investigation on Echinococcus infection in cattle and sheep in Qumalai County. Qinghai Journal of Animal and Veterinary Sciences, 41(05), 65. (In Chinese)

Yuan, Z.Y. (2015) Investigation on echinococcosis in cattle and sheep in Xinghai County, Qinghai Province. Shangdong Journal of Animal Science and Veterinary Medicine, 36(04), 52. (In Chinese)

Zhou, M. and Yang, J.P. (2009) Investigation on Echinococcus infection in slaughtered cattle in Jiuzhi County. Qinghai Journal of Animal and Veterinary Sciences, 39(04), 29. (In Chinese)

Zhou, M.C. (2018) Investigation on echinococcosis in cattle and sheep in Maqin County. Chinese Abstracts of Animal Husbandry and Veterinary Medicine, 34(02),123. (In Chinese)
